# The effect of lncRNA MIR155HG-modified MSCs and exosome delivery to synergistically attenuate vein graft intimal hyperplasia

**DOI:** 10.1186/s13287-022-03197-0

**Published:** 2022-11-04

**Authors:** Xiao Bai, Zaiwen Qi, Mingzhen Zhu, Zhuangzhuang Lu, Xin Zhao, Lining Zhang, Guangmin Song

**Affiliations:** 1 Department of Cardiovascular Surgery, Qilu Hospital of Shandong University, Jinan, 250012 China; 2 Thoracoscopy Institute of Cardiac Surgery, Shandong University, Jinan, China; 3 The Fifth People’s Hospital of Jinan, Jinan, China; 4 Department of Immunology, School of Basic Medical Science, Shandong University, Jinan, 250012 China

**Keywords:** MIR155HG, Mesenchymal stem cells, Exosome, Vein graft, Intimal hyperplasia

## Abstract

**Background:**

The mesenchymal stem cells (MSCs) were used to repair tissue injury. However, the treatment effect was not satisfactory. We investigated whether lncRNA MIR155HG could promote survival and migration of MSCs under oxidative stress, which mimics in vivo environments. Furthermore, we studied the protective effect of exosomes secreted by MSCs transfected with MIR155HG on endothelial cells. This study aimed to determine whether exploiting MSCs and exosomes modified with lncRNA MIR155HG would exert synergistic therapeutic effect to attenuate vein graft intimal hyperplasia more effectively.

**Methods:**

Lentivirus containing lncRNA MIR155HG overexpressing vector was packaged and used to infect MSCs. Then, CCK-8 assay, flow cytometry, Transwell assay, and Elisa assay were used to assess the functional changes of MSCs with overexpressed MIR155HG (OE-MSCs). Furthermore, the associated pathways were screened by Western blot. MIR155HG-MSCs-derived exosomes (OE-exo) were collected and co-cultured with human umbilicus vein endothelial cell (HUVEC). We validated the protective effect of OE-exo on HUVEC. In vivo, both MSCs and exosomes modified with MIR155HG were injected into a vein graft rat model via tail vein. We observed MSCs homing and intimal hyperplasia of vein graft using a fluorescent microscope and histological stain.

**Results:**

Our study found that lncRNA MIR155HG promoted proliferation, migration, and anti-apoptosis of MSCs. NF-κB pathway took part in the regulation process induced by MIR155HG. OE-exo could enhance the activity and healing ability of HUVEC and reduce apoptosis. In vivo, OE-MSCs had a higher rate of homing to vascular endothelium. The combined treatment with OE-MSCs and OE-exo protected vascular endothelial integrity, reduced inflammatory cell proliferation, and significantly attenuated intimal hyperplasia of vein graft.

**Conclusion:**

LncRNA MIR155HG could promote the survival and activity of MSCs, and reduce the apoptosis of HUVECs using exosome delivery. Exploiting MSCs and exosomes modified with MIR155HG could attenuate vein graft intimal hyperplasia more effectively and maximize the surgical effect.

## Background

Coronary artery bypass grafting (CABG) is the most effective treatment for severe coronary heart disease. The autogenous veins are commonly used as bridging vessels in CABG. However, approximately 20–40% of vein grafts could become stenosed or even occluded two years after surgery [1, 2]. Intimal hyperplasia of vein graft seriously reduces the therapeutic effect of CABG [3, 4]. The pathological basis of intimal hyperplasia is endothelial cell injury [5], which induces oxidative stress reaction and delays vascular endothelial repair [6]. These pathological processes lead to intimal hyperplasia, lumen stenosis, and occlusion. Therefore, early repair of the vascular endothelium is essential in preventing graft remodeling [7, 8].

The mesenchymal stem cells (MSCs) are used to repair tissue injury [9, 10], and endothelial cells serve as a vascular barrier. The apoptosis of endothelial cells would destroy the barrier, directly exposing vascular smooth muscle cells to blood flow, leading to the inflammatory hyperplasia. Our previous studies confirmed that MSCs could be home in the endothelial injury location to promote endothelial repair, attenuating vein graft intimal hyperplasia. However, studies suggested a high level of oxidative stress caused by vein grafting in the blood circulation. The survival and activity of MSCs could be decreased under oxidative stress induced by tissue injury, weakening the effect of MSCs therapy [11]. Therefore, we enhanced the function of MSCs in the oxidative stress environment using long non-coding RNA (lncRNA) to improve the therapeutic effect of MSCs transplantation.

lncRNA is a non‐coding RNA with more than 200 nucleotides in length. lncRNA MIR155 host gene (MIR155HG), also known as B‐cell integration cluster, located in chromosome 21q21, is considered the precursor of miR-155 [12, 13]. MIR155HG could promote the migration and invasion of cervical cancer cells [14], and MIR155HG knockdown suppresses cell proliferation, migration, and invasion in non-small cell lung cancer [15]. There is no evidence that MIR155HG effects migration and the anti-apoptotic ability of MSCs. In this study, we try to use MIR155HG to improve the migration and anti-apoptotic ability of MSCs. The increase in the homing MSCs in vivo may promote endothelial repair effect and better alleviate intimal hyperplasia of grafted veins.

The exosomes secreted by MSCs are tiny membranous vesicles with lipid bilayers, 30–150 nm in diameter. Exosomes are rich in lncRNA, miRNA, and other genetic materials [16, 17] and worth studying their functional changes after MIR155HG transfection. Exosomes downregulate inflammatory response and reduce apoptosis [18, 19]. Therefore, we want to study the change of genetic materials in the exosomes derived from MIR155HG-MSCs and the protective effect of exosomes on endothelial cells.

In this study, we attenuated vein graft intimal hyperplasia by exploiting both MSCs and exosomes modified with MIR155HG. We showed that MIR155HG promoted the migration and anti-apoptosis of MSCs. MIR155HG-exosomes enhanced the healing ability of HUVEC and reduced the apoptosis. MIR155HG could serve as a target for modifying MSCs and exosomes to prevent intimal hyperplasia of vein graft and improve the effect of vascular transplantation.

## Materials and methods

### Cell culture and characterization

The MSCs were purchased from Cyagen Biosciences Inc. (Shanghai, China) and were cultured in DMEM containing 10% fetal bovine serum (Gibco, USA) in a humidified atmosphere at 37 °C and 5% CO_2_. MSCs were characterized by cell surface markers (CD29, CD34, CD45 and CD90) using flow cytometric analysis. The HUVECs were purchased from FuHeng Biology (Shanghai, China) and were cultured in ECM (ScienCell, USA) containing 5% fetal bovine serum and 1% endothelial cell growth supplements. Cells were maintained at 37 °C in a humidified atmosphere of 5% CO_2_. HUVECs were characterized by CD31.

### Lentiviral vector transfection

The MIR155HG-expressing lentivirus vector was pCDH-CMV-MIR155HG-EF1-copGFP-T2A-Puro(rLv-MIR155HG) obtained from OLIGOBIO Co., Ltd. (Beijing, China). The rLv-MIR155HG vector and control vector were, respectively, co-transfected with packaging vectors PCDH-CMV-MCS-EF1-copGFP-T2A-puro, psPAX2 and p MD2.G into HEK-293T cells using Lipofectamine 2000 transfection reagent. The MIR155HG-knockdown lentivirus vector was plvx-shRNA2-Zsgreen-T2A-puro. Short-hairpin RNAs (shRNAs) against MIR-155HG were constructed in pcDNA3.1 by OLIGOBIO Co., Ltd. (Beijing, China). The target sequence of sh-MIR155HG was GCATTCACGTGGAACAAAT, and the control sequence was TTCTCCGAACG- TGTCACGT. Primary MSCs were incubated with recombinant MIR155HG-GFP or sh-MIR155HG-GFP lentivirus vectors at a multiplicity of infection (MOI) of 60. Cells were infected with the lentivirus medium. After 6 h, 2 ml fresh medium was added to dilute polybrene. Then, the lentivirus culture medium was replaced with a fresh medium for another 24 h. The green fluorescent protein signal was detected using a fluorescence microscope, and gene transfection efficiency was verified using PCR 48 h later. Primers were as follows: MIR155HG forward 5′-GCTTGCTGAAGGC TGTATGC-3′, MIR155HG reverse 5′-GTCTTGTCATCCTCCCACGG-3′; GAPDH forward 5′-GATTTGGCCGTATCGGAC-3′, GAPDH reverse 5′-GAAGACGCCA GTAGACTC-3′. Each reaction was replicated three times. Fold changes in cDNA relative to GAPDH endogenous control were calculated using the 2^−ΔΔCt^ method.

### Chemical treatment

NF-κB inhibitor BMS-345541 was purchased from MCE (New Jersey, USA). Cells were treated with 5 μM BMS-345541 for 2 h. The NF-κB pathway was significantly inhibited at this concentration, but no apoptosis was observed.

### Cell viability assay

Cell viability was assessed using a Cell Counting Kit-8 (CCK-8) (MCE, New Jersey, USA). Cells were seeded onto 96 well plates and incubated for 24, 48, 72, and 96 h. Then, 10% CCK-8 solution was added to each well according to the manufacturer’s instructions. Two hours later, the absorbance at 450 nm was detected using enzyme micro-plate reader (Tecan F50, Switzerland).

### Cell proliferation assay

Cell proliferation was assessed using a kFluor555 Click-iT EdU Kit (Keygen Biotech, Nanjing, China) according to the manufacturer’s instructions. KFluor555 stained the proliferating nuclei in red, and Hoechst 33342 stained the nucleus in purple. The proliferation rate equals the number of cells in proliferation state (red) divided by the number of total cells (purple).

### Quantitative real-time PCR (qRT-PCR)

Total RNA was isolated from samples using TRIzol reagent (Invitrogen, USA), and reverse transcription was performed using the PrimeScript RT reagent kit (Takara, Japan). QRT-PCR with SYBR Green was performed using a Bio-Rad real-time PCR system according to the manufacturer’s protocol. Melt curve analysis was conducted to verify that only one product was produced. RNA levels were calculated relative to GAPDH levels using the 2^−ΔΔCt^ method.

### Western blot

Proteins were subjected to SDS-PAGE on polyacrylamide gels (8–10%) and transferred onto a PVDF membrane. After blocking with 5% non-fat milk in TBS containing 0.1% Tween-20, the membrane was incubated at 4 °C overnight with one of the following primary antibodies: anti-p-NF-κB P65, anti-NF-κB P65, anti-p-mTOR, anti-p-ERK, anti-ERK, anti-PDCD4, anti-GNA12 (Affinity, USA); anti-mTOR, anti-GAPDH, anti-TSG101, anti-CD63, anti-CD81, anti-Bax (Proteintech, USA); anti-Bcl-2 (Abclonal, China). Subsequently, the peroxidase-conjugated AffiniPure goat anti-rabbit or mouse IgG (Proteintech, USA) was added. Bound antibody was visualized via ECL plus TM Western blotting system detection kit (Amersham, USA).

### Migration assay

A cell migration assay was performed using a transwell (8 μm pore size) (Corning, USA) to observe the migration function of MSCs. 200 μL transfected MSCs (1.5 × 10^5^/ml) were seeded in the upper chamber, and 600 μl complete medium with SDF-1a (100 ng/ml, Proteintech, USA) were placed into the lower chamber. Cells on the upper side of the membrane were removed after 12 h. Cells on the bottom surface of the membrane were stained with 0.1% crystal violet and counted in 5 randomly selected microscopic fields.

### Cell-surface phenotype analysis

Experimental groups of MSCs were stained with rabbit anti-rat CXCR4 antibody (1:250, abcam, UK). The blank control group was stained with the isotype control antibody. Cells were incubated at 4 °C for 1 h. Then, the secondary antibody Cy3 goat anti-rabbit IgG (H + L) (Abclonal, China) was added and incubated at 4 °C for 20 min. Flow cytometry was used to identify the phenotypes of MSCs.

### Cell apoptosis

The flow cytometry was performed using an Annexin V/PI apoptosis detection kit (Keygen Biotech, Nanjing, China) to quantify the apoptosis of the MSCs according to the manufacturer’s instructions.

### ELISA assay

The culture medium of each group of MSCs was collected and centrifuged at 1000 g for 20 min to obtain the supernatant. The HGF or VEGF concentration in the supernatant was quantified using an enzyme-linked immunosorbent assay (ELISA) kit (ExCellBio, Shanghai, China).

### Extraction and identification of exosomes derived from MSCs

Exosomes were extracted from supernatants of MSCs cultures using density gradient ultracentrifugation. Morphology of exosomes was observed using a transmission electron microscope after uranyl acetate staining. Exosome particle size was detected using a nanometer particle size detector. The markers of exosomes, including CD63, CD81, and TSG101, were identified using western blot. Uptake of exosome by HUVECs was observed using confocal laser microscopy (Leica, Germany).

### Vein grafting models

All the Sprague–Dawley (SD) rats weighing 250–300 g were purchased from Charles River (Beijing, China). The external jugular vein was harvested without damage from the neck of the rats. Small branches of blood vessels were ligated using thin silk threads. The length of the obtained vein was approximately 1.5 cm. Vein grafting was performed with the cuff technique and inserted into the infrarenal abdominal aorta in the same rat. Heparin (100U/100 g) was used before and after grafting. Transfected cells (5 × 10^7^/ml × 0.2 ml) and exosome (400 μg protein suspended in 0.2 ml PBS) were injected into rat model through the caudal vein 24 h after grafting. Rats were humanely killed one week after operation to evaluate MSC homing using fluorescence microscopy. All remaining rats were killed four weeks after the operation for histological detection. All the experimental procedures were performed with the approval of the Ethical Committee of the Qilu Hospital of Shandong University (KYLL-2021(KS)-976) and followed the Institutional Animal Care and Use Committee guidelines.

### Histological examination

Vascular specimens were fixed in 4% paraformaldehyde and then embedded in paraffin. Paraffin specimen sections (4-μm-thick) were prepared by dewaxing. Van Gieson (VG) stain was used to assess collagen infiltration according to the manufacturer’s instruction. The morphology of the vascular intima was observed using light microscopy. The nucleus was stained in blue, muscle fibers were stained in yellow, and collagen fibers appeared bright red.

For the immunohistochemistry test, the sections were incubated with rabbit anti-rat PCNA (1:100) or NF-κB P65 (1:100) overnight at 4℃. Then, sections were incubated with biotinylated goat anti-rabbit IgG (1:200) for 30 min. Positive staining was identified using diaminobenzidine (DAB). Positive staining for PCNA or NF-κB P65 was observed as brown areas. We applied Image J software to calculate the integral optical density (IOD) of each field.

### Immunofluorescence microscopy

First, the sections were stained with anti-CD31 (Affinity, USA) overnight at 4°C. The secondary antibody was added after the primary antibody was washed with PBS. Sections were incubated for 45 min at room temperature. Nuclei were counterstained with DAPI. Images were observed using a fluorescent microscope (OLYMPUS, Japan).

### Statistical analysis

Data were expressed as means and standard deviations (SD). SPSS version 20.0 software (SPSS, Chicago, USA) was used to analyze differences between samples, either using the two-sample student *t* test or one-way ANOVA for differences between selected pairs of samples. *P* value < 0.05 was considered statistically significant.

## Results

### Characterization of MSCs and lentiviral transfection of lncRNA MIR155HG

**Fig. 1:**
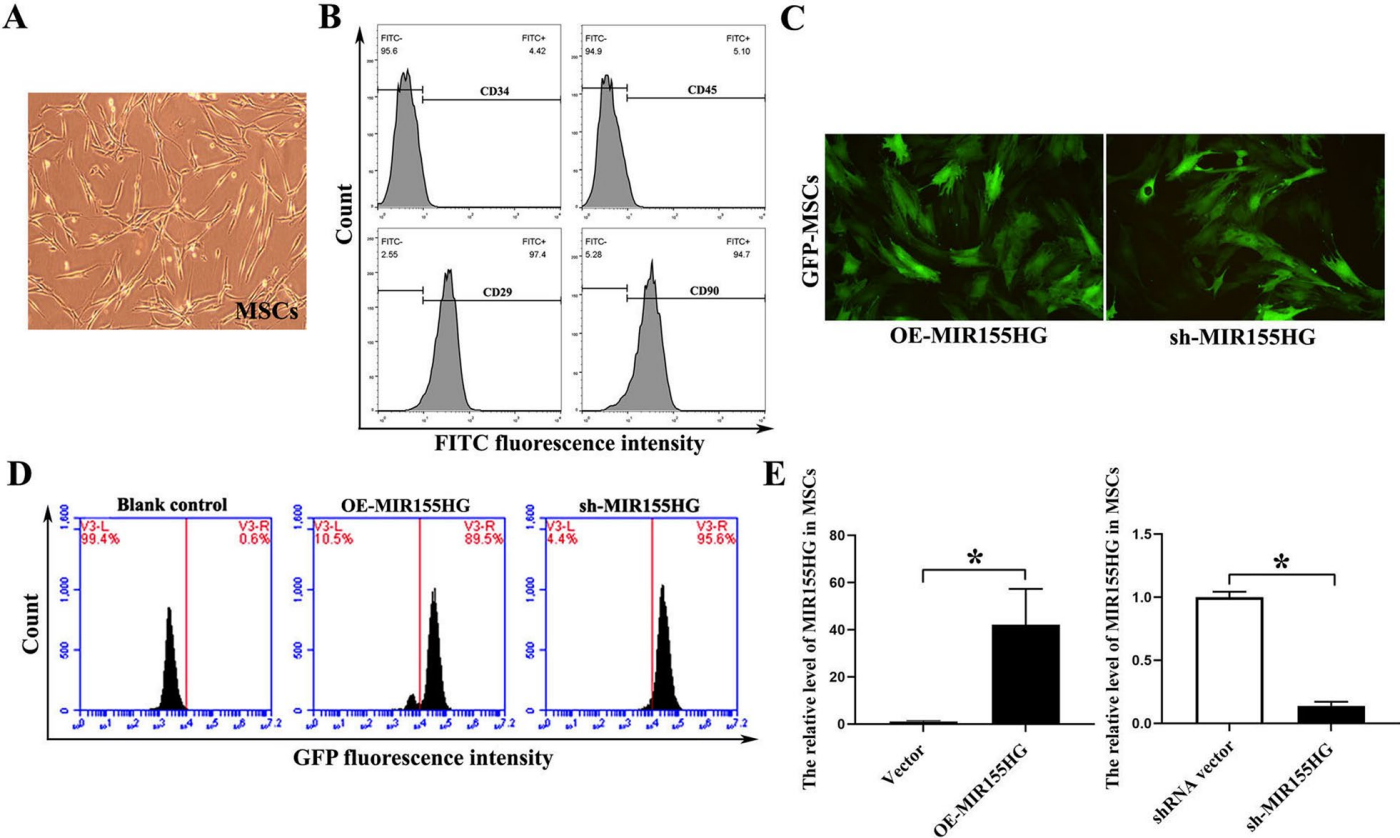
Characterization of MSCs and lentiviral transfection. **A** Cultured MSCs exhibited a fibroblast-like morphology. **B** Flow cytometric analysis of cultured cells with CD29, CD34, CD45, and CD90 anti-bodies. Results validated the mesenchymal origin of the cell population used in our study. **C** GFP-labeled MSCs showed green fluorescence under a fluorescent microscope. **D** Transfection efficiency of overexpression and knockdown lentivirus were qualified. **E** RT-PCR was used to evaluate the effect of MIR155HG overexpression and knockdown

The MSCs cultured in vitro presented a homogeneous fibroblast-like form (Fig. 1A). Flow cytometry demonstrated that the cells were uniformly negative for the hematopoietic markers CD34 and CD45 and positive for the stem cell antigens CD29 and CD90 (Fig. 1B). Thus, the phenotype of the cell population used in our study was consistent with that of MSCs. MSCs were transfected with recombinant MIR155HG-GFP or sh-MIR155HG-GFP lentiviral vector (MOI = 60). GFP-labeled MSCs showed strong green fluorescence under a fluorescent microscope (Fig. 1C). GFP fluorescence intensity was detected by flow cytometry, and the result showed that the transfection efficiency of lentivirus to MSCs was approximately 90% (Fig. 1D). The high transfection efficiency could meet the experimental requirements. After transfection with MIR155HG, the level of MIR155HG in the OE-MIR155HG group was 40.14 times that in the vector group. After MIR155HG interfered with shRNA, the level of MIR155HG in the sh-MIR155HG group was 0.14 times that in the shRNA vector group (Fig. 1E). The overexpression or knock-down of MIR155HG was successfully achieved in this experiment.

### MIR155HG regulates MSCs through the NF-κB pathway

**Fig. 2:**
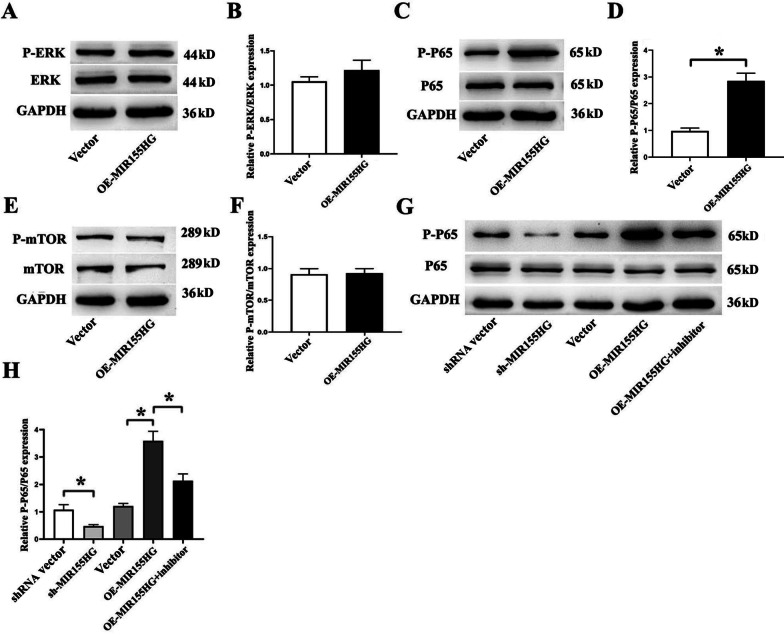
MIR155HG regulates MSCs through the NF-κB pathway. **A** Western blot analysis of P-ERK and ERK. **B** There was no significant difference in P-ERK/ERK expression between the two groups. **C** Western blot analysis of P-P65 and P65. **D** Phosphorylation level of p65 was significantly increased by MIR155HG overexpression (*p < 0.05). **E** Western blot analysis of P-mTOR and mTOR. **F** There was no significant difference in P-mTOR/mTOR expression between the two groups. **G** Western blot analysis of phosphorylation levels of P65 with or without NF-κB inhibitor BMS-345541. **H** Phosphorylation level of P65 was significantly decreased by sh-MIR155HG. BMS-345541 abolished the increased level of P-P65 caused by MIR155HG overexpression (*p < 0.05)

We attempted to explore the molecular changes in MSCs under the impact of MIR155HG overexpression. According to literature and our previous studies, several pathway proteins, including ERK, NF-κB p65, and mTOR, and their phosphorylated forms were screened out for the test. These pathways were widely involved in cell activity, apoptosis, tumor metastasis, and angiogenesis. As indicated by the western blot, there was no significant change in the level of P-ERK/ERK (Fig. 2A, B) or P-mTOR/mTOR (Fig. 2E, F). However, the phosphorylation level of NF-κB p65 was significantly increased by MIR155HG overexpression (*p* < 0.05, Fig. 2C, D).

The OE-MIR155HG group was treated with BMS-345541 (NF-κB inhibitor, 5 μM) to confirm that MIR155HG could regulate the NF-κB pathway in MSCs. BMS-345541 abolished the increased phosphorylation level of p65 caused by OE-MIR155HG (Fig. 2G, H). These results indicated that MIR155HG could regulate MSCs through the NF-κB pathway.

### MIR155HG promotes the proliferation and migration of MSCs via the NF-κB pathway

**Fig. 3:**
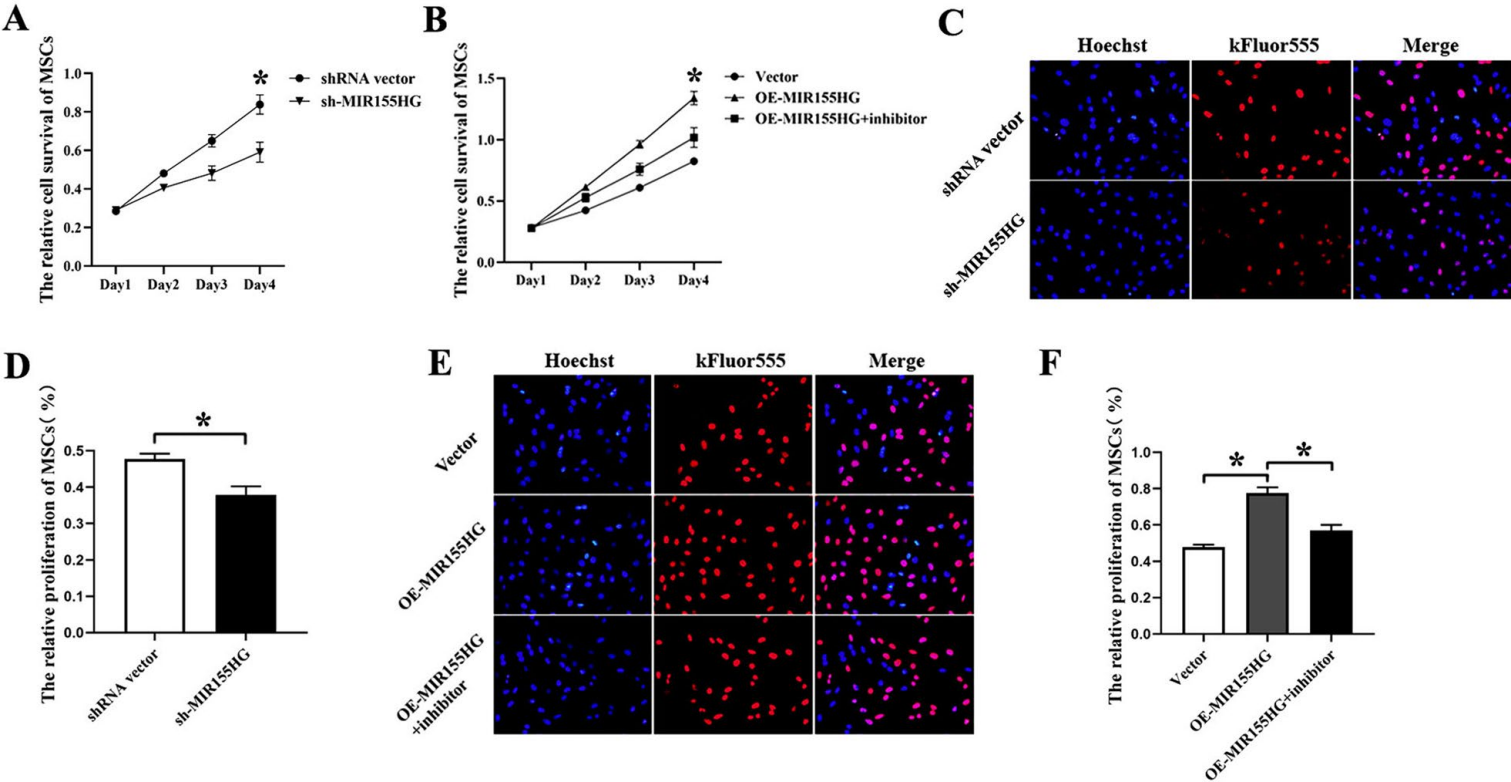
Effects of MIR155HG on cell viability and proliferation in MSCs. **A** sh-MIR155HG reduced cell viability significantly (*p < 0.05). **B** OE-MIR155HG promoted the viability of MSCs, and the NF-κB inhibitor could counteract that effect (*p < 0.05). **C**, **D** EDU assay showed that sh-MIR155HG inhibited the proliferation of MSCs (*p < 0.05). **E**, **F** OE-MIR155HG promoted the proliferation of MSCs, and the NF-κB inhibitor could counteract that effect (*p < 0.05)

CCK-8 assay was applied to study the effect of MIR155HG on the viability of MSCs. The results indicated that sh-MIR155HG significantly inhibited the viability of MSCs (Fig. 3A). OE-MIR155HG increased total cell viability, which was reduced by BMS-345541 antagonizing the NF-κB pathway (Fig. 3B). The EdU assay suggested that sh-MIR155HG significantly inhibited the proliferation of MSCs (Fig. 3C, D). OE-MIR155HG promoted cell proliferation significantly. Besides, BMS-345541 could reverse that role, which confirmed that the NF-κB pathway was involved in the regulatory process (Fig. 3E, F).

**Fig. 4:**
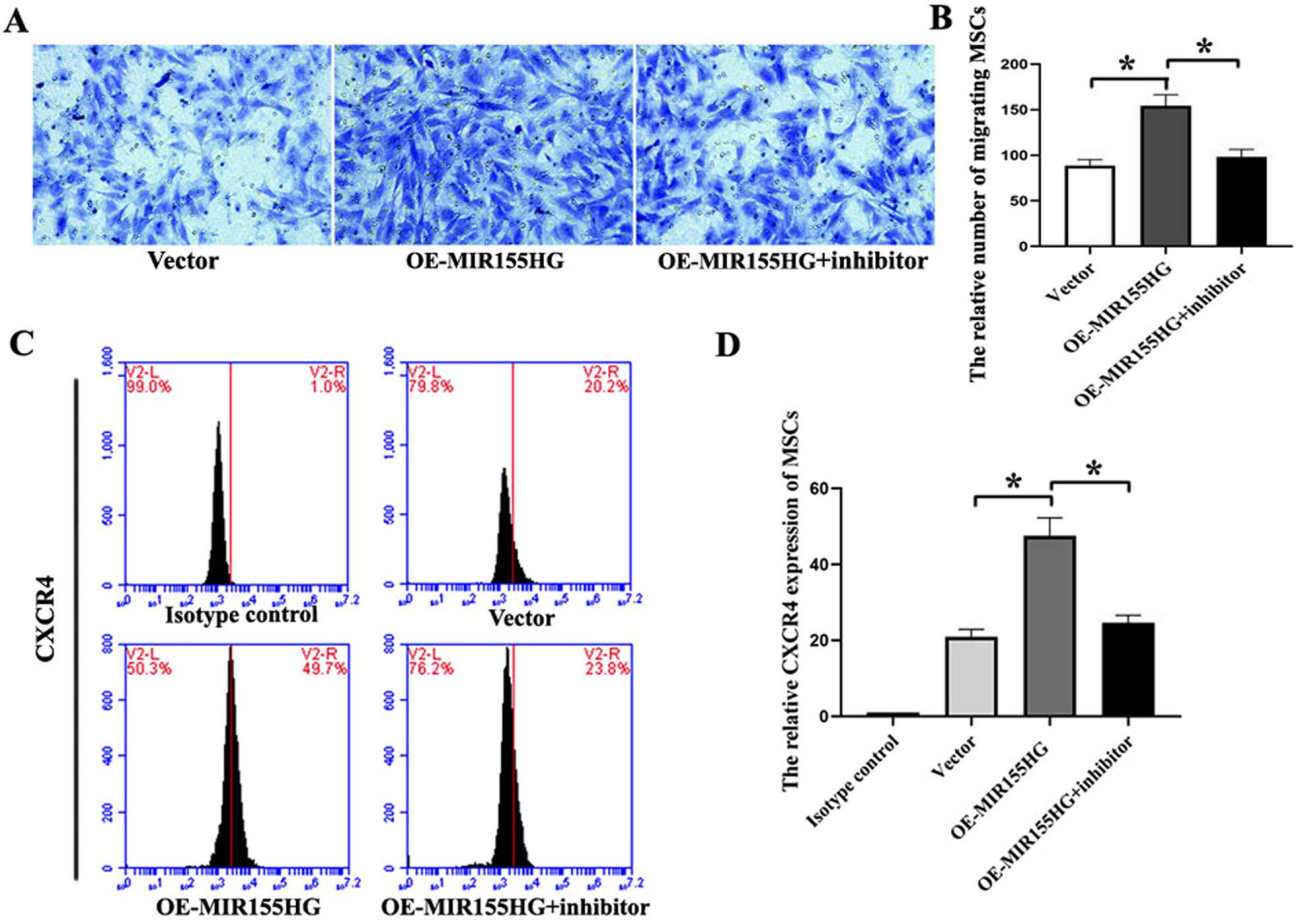
Effects of MIR155HG on migration in MSCs. **A** Representative transwell images of MSCs in 3 groups. **B** OE-MIR155HG promoted the migration of MSCs, and BMS-345541 could reverse that role (*p < 0.05). **C**, **D** The CXCR4 expression rate on the surface of MSCs was analyzed by flow cytometry. OE-MIR155HG promoted the expression of CXCR4 (*p < 0.05)

In addition, we observed the effect of MIR155HG on migration of MSCs. Transwell assay showed that OE-MIR155HG promoted the migration of MSCs, and NF-κB inhibitor could block the effect. NF-κB pathway regulated MIR155HG on the migration of MSCs (Fig. 4A, B). Flow cytometry indicated that CXCR4 expressed on the surface of MSCs was increased following OE-MIR155HG (Fig. 4C, D), causing enhanced migration of MIR155HG-MSCs. Several articles suggested that CXCR4 was the primary molecule mediating MSCs migration [20, 21].

### MIR155HG reduces oxidative stress-induced apoptosis in MSCs via the NF-κB pathway

**Fig. 5:**
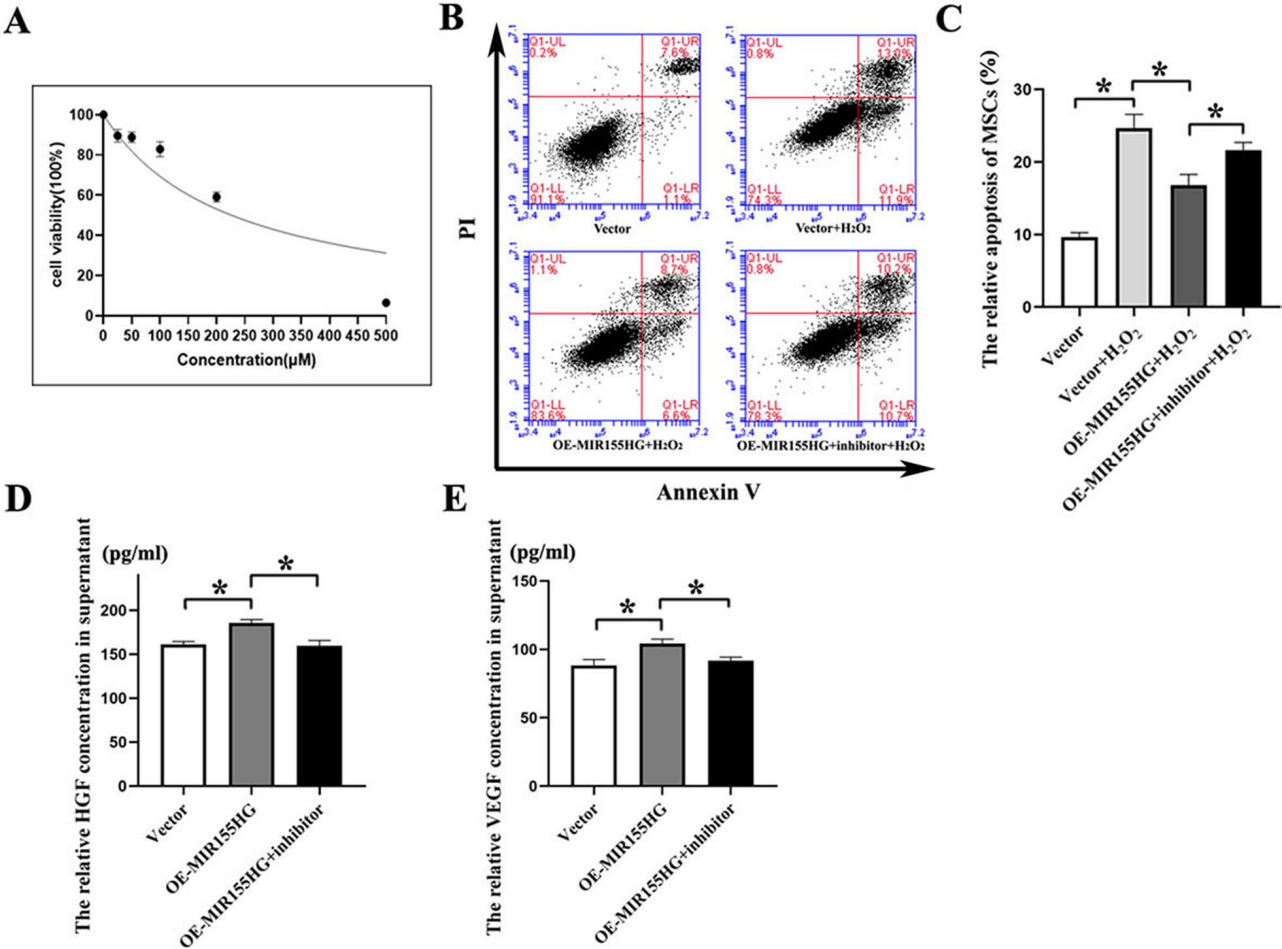
The anti-apoptosis role of MIR155HG on MSCs under oxidative stress. **A** CCK-8 assay displayed that the IC50 value of H_2_O_2_ against MSCs was 226.5 μM. **B**, **C** Flow cytometry revealed that OE-MIR155HG reduced oxidative stress-induced apoptosis in MSCs. NF-κB inhibitor could counteract that protective effect of MIR155HG on MSCs (*p < 0.05). **D**, **E** ELISA assay showed that MIR155HG promoted the secretion level of HGF and VEGF in the supernatant of MSCs (*p < 0.05)

We used hydrogen peroxide to simulate oxidative stress. Following treatment with H_2_O_2_ of different concentrations for 12 h, the viability of MSCs was markedly decreased. CCK-8 assay displayed that the half-maximal inhibitory concentration (IC50) was 226.5 μM (Fig. 5A). Therefore, we chose 220 μM as the experimental concentration. Flow cytometry demonstrated that H_2_O_2_-induced oxidative stress led to significant apoptosis of MSCs. OE-MIR155HG could effectively relieve the apoptosis caused by oxidative stress. The protective role of OE-MIR155HG was reversed by BMS-345541, antagonizing the NF-κB pathway (Fig. 5B, C).

MSCs could secrete anti-apoptotic factors, such as hepatocyte growth factor (HGF) and vascular endothelial growth factor (VEGF). Elisa assay showed that OE-MIR155HG significantly elevated the level of HGF and VEGF in supernatant (Fig. 5D, E). MIR155HG regulated the secretion of pro-survival factors via the NF-κB pathway.

### OE-exosome could deliver lncRNA MIR155HG into HUVECs

**Fig. 6:**
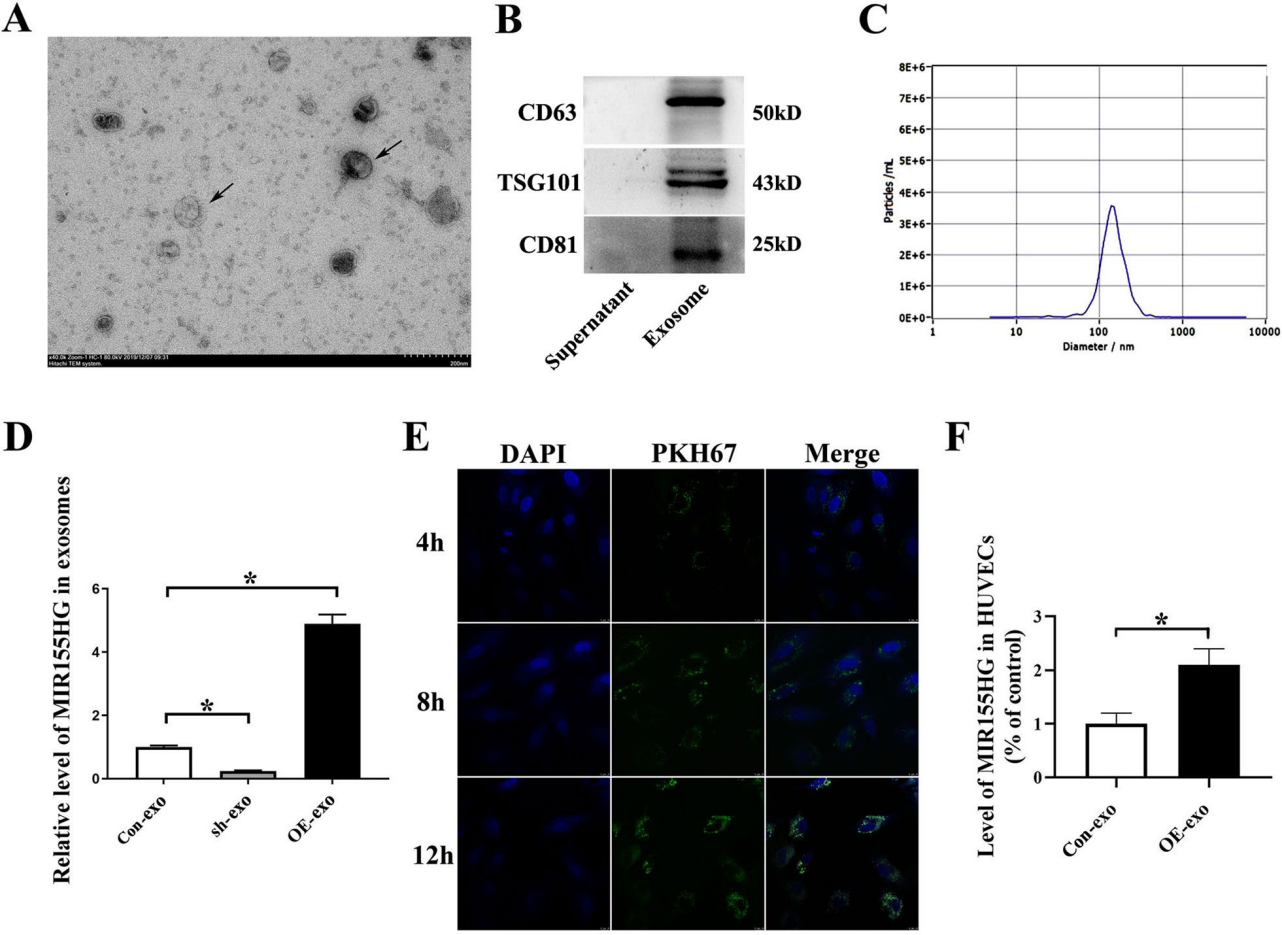
Characterization of exosomes derived from MSCs. **A** Electron microscope image of exosomes stained with uranyl acetate. The black arrow points to exosomes. **B** Exosomes were identified using CD63, TSG101, and CD81. **C** Particle diameter of the exosome was approximately 143.6 nm. **D** RT-PCR detected the level of MIR155HG in exosomes derived from sh-MSCs or OE-MSCs (*p < 0.05). **E** Exosomes were stained in green using PKH67. Exosomes could be taken up apparently by HUVECs after 12 h. **F** The MIR155HG level in HUVECs after exosome ingestion (*p < 0.05)

Exosomes were extracted from the culture medium of MSCs. The exosome particles were typical saucer-like shape under the transmission electron microscope (Fig. 6A). Western blot was used to identify exosome-specific phenotypic markers CD63, CD81 and TSG101 on the surface of the vesicles (Fig. 6B). Detection of nanoparticle size showed that the particle diameter was approximately 143.6 nm, consistent with exosome size (30–200 nm) (Fig. 6C). These results were consistent with previous reports, and these vesicles were confirmed as exosomes of MSCs origin.

Exosomes are capable of delivering genetic materials. Based on qRT-PCR, we observed that the RNA level of MIR155HG in exosomes derived from MIR155HG-MSCs was 4.9-fold higher than that from control-MSCs (Fig. 6D). A similar genetic material change was observed in exosomes as in transfected MSCs. In the co-incubation experiment, we observed that PKH67-labeled exosomes could fuse with the cell membrane and get inside HUVECs. Exosomes could be taken up apparently by HUVECs after 12 h (Fig. 6E). Furthermore, the level of MIR155HG in HUVECs treated with MIR155HG-exosomes (OE-exo) was 2.1-fold higher than that with control-exosomes (Con-exo) (Fig. 6F). The results confirmed that exosomes derived from OE-MSCs could deliver MIR155HG into HUVECs.

### OE-exo promotes migration and reduces apoptosis of HUVECs

**Fig. 7:**
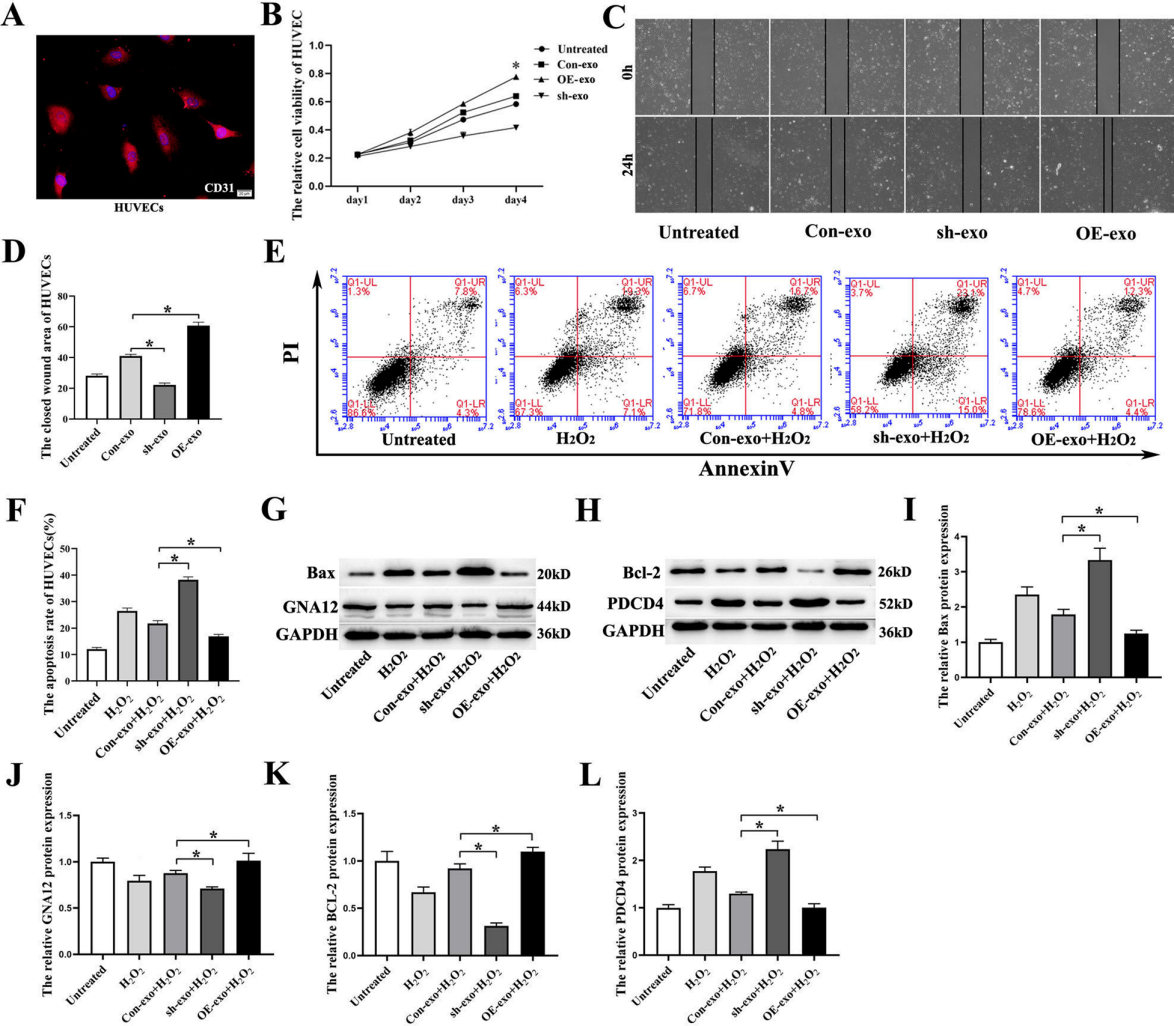
Functional effects of OE-exo on HUVECs. **A** HUVECs were immune-stained with CD31 (red) and DAPI (purple). **B** CCK-8 assay showed that OE-exo promoted the viability of HUVECs, and sh-exo reduced cell viability (*p < 0.05). **C**, **D** OE-exo promoted migration of HUVECs, and sh-exo inhibited cell migration as demonstrated using wound healing assay (*p < 0.05). **E**, **F** Flow cytometry revealed that OE-exo reduced apoptosis of HUVECs induced by oxidative stress, and sh-exo aggravated apoptosis of HUVECs (*p < 0.05). **G**, **H** Representative western blots of Bax, GNA12, Bcl-2, and PDCD4 protein expression in HUVECs. **I**–**L** OE-exo up-regulated the expression of GNA12 and Bcl-2 in HUVECs. OE-exo reduced the expression of Bax and PDCD4 in HUVECs (*p < 0.05)

HUVECs were identified using CD31 staining (Fig. 7A). CCK-8 assay was used to investigate the effect of OE-exo on the cell viability of HUVECs. Compared with the Con-exo group, the activity of HUVECs in OE-exo group was significantly enhanced. The activity of HUVECs in the sh-exo group was reduced (Fig. 7B). In addition, we used a wound healing assay to observe the function of OE-exo on the migration of HUVECs. The results showed that OE-exo could promote the migration of HUVECs and accelerate endothelial healing (Fig. 7C, D).

The flow apoptosis assay showed that OE-exo significantly alleviated apoptosis of HUVECs caused by oxidative stress. However, the apoptosis was increased in the sh-exo group (Fig. 7E, F). We used western blot to investigate the molecular mechanism that led to these effects in HUVECs. The results showed that two anti-apoptotic proteins, including GNA12 and Bcl-2, were up-regulated in the OE-exo group. Furthermore, the pro-apoptotic proteins, including Bax and PDCD4, were down-regulated (Fig. 7G–L).

### Combined use of OE-MSCs and OE-exo significantly attenuates vein graft intimal hyperplasia

**Fig.8:**
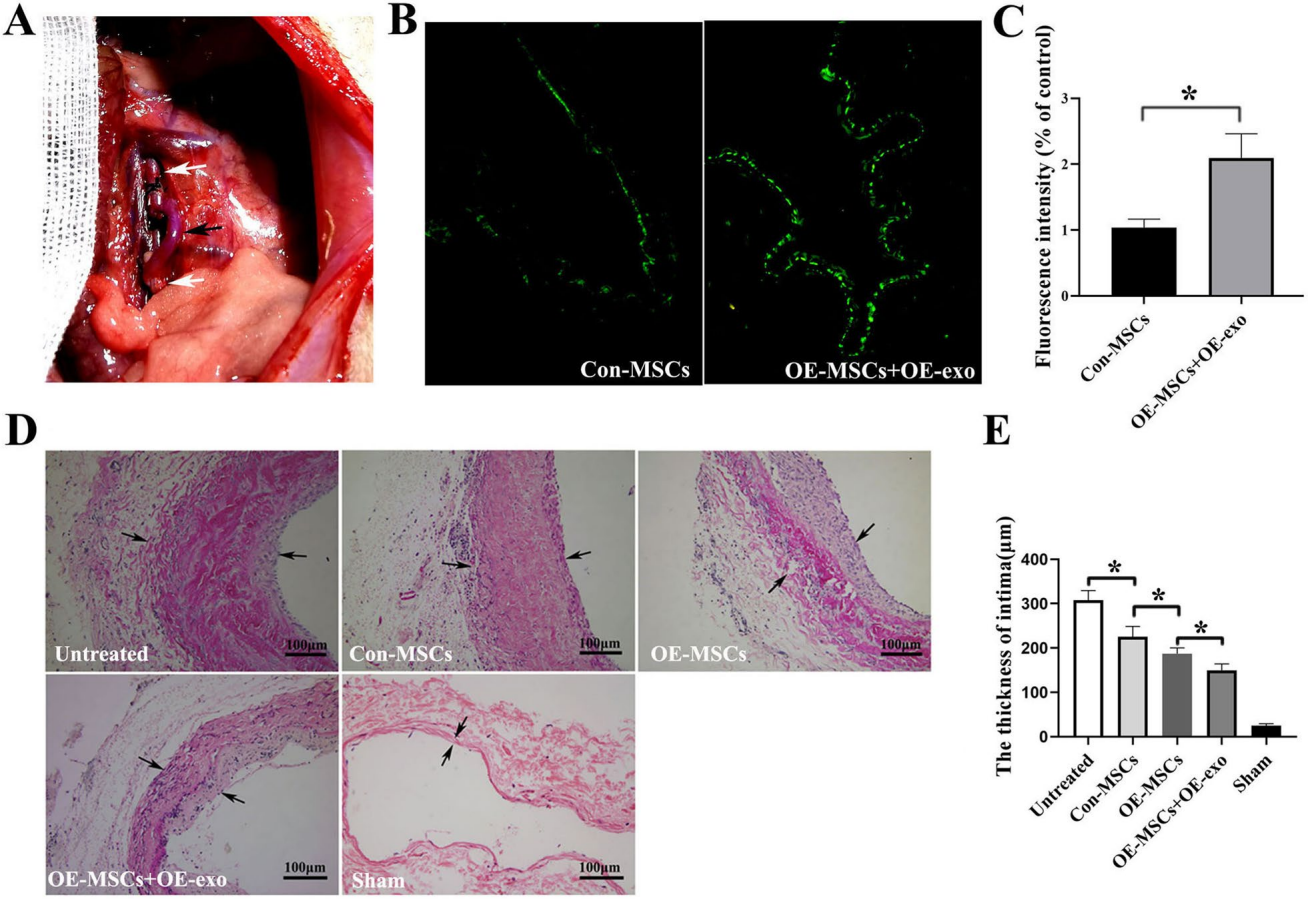
Evaluation of MSCs homing and intimal thickness of vein graft. **A** Rat model of autogenous vein graft was established. The black arrow indicated the grafted vein, and the white arrow represented the abdominal aorta. **B** Representative images of GFP-MSCs homing to the graft vein endothelium. MSCs carrying GFP showed a green light under the fluorescence microscope. **C** Fluorescence intensity of homing MSCs was quantitatively analyzed (*p < 0.05). **D** Representative Van Gieson staining images of vein graft. The black arrow indicated the intima of the vein graft. **E** Quantitative analysis of vein graft intimal thickness (*p < 0.05)

The autologous external jugular vein-abdominal aorta transplantation model in rats used in this experiment was skilled grasping. The external jugular vein was inserted into the infrarenal abdominal aorta in the same rat with the cuff technique (Fig. 8A). The model could well simulate autogenous vein intima hyperplasia clinically after coronary artery bypass graft (CABG). After transfected MSCs and exosomes were injected into the rat model through the caudal vein, the fluorescence microscope was used to observe the homing of MSCs to vein graft. The number of OE-MSCs migrated to the intima of vein graft was significantly increased compared to the control group (Fig. 8B, C). VG staining was performed to assess neointimal hyperplasia. The collagen fibers were stained in red, and the muscles were stained in yellow. The results demonstrated that neointima of the OE-MSCs group was thinner than that of the Con-MSCs group. The effect of the OE-MSCs + OE-exo group was better than that of the OE-MSCs group. Intimal hyperplasia in the grafted vein was significantly reduced in the OE-MSCs + OE-exo group (Fig. 8D, E).

**Fig. 9:**
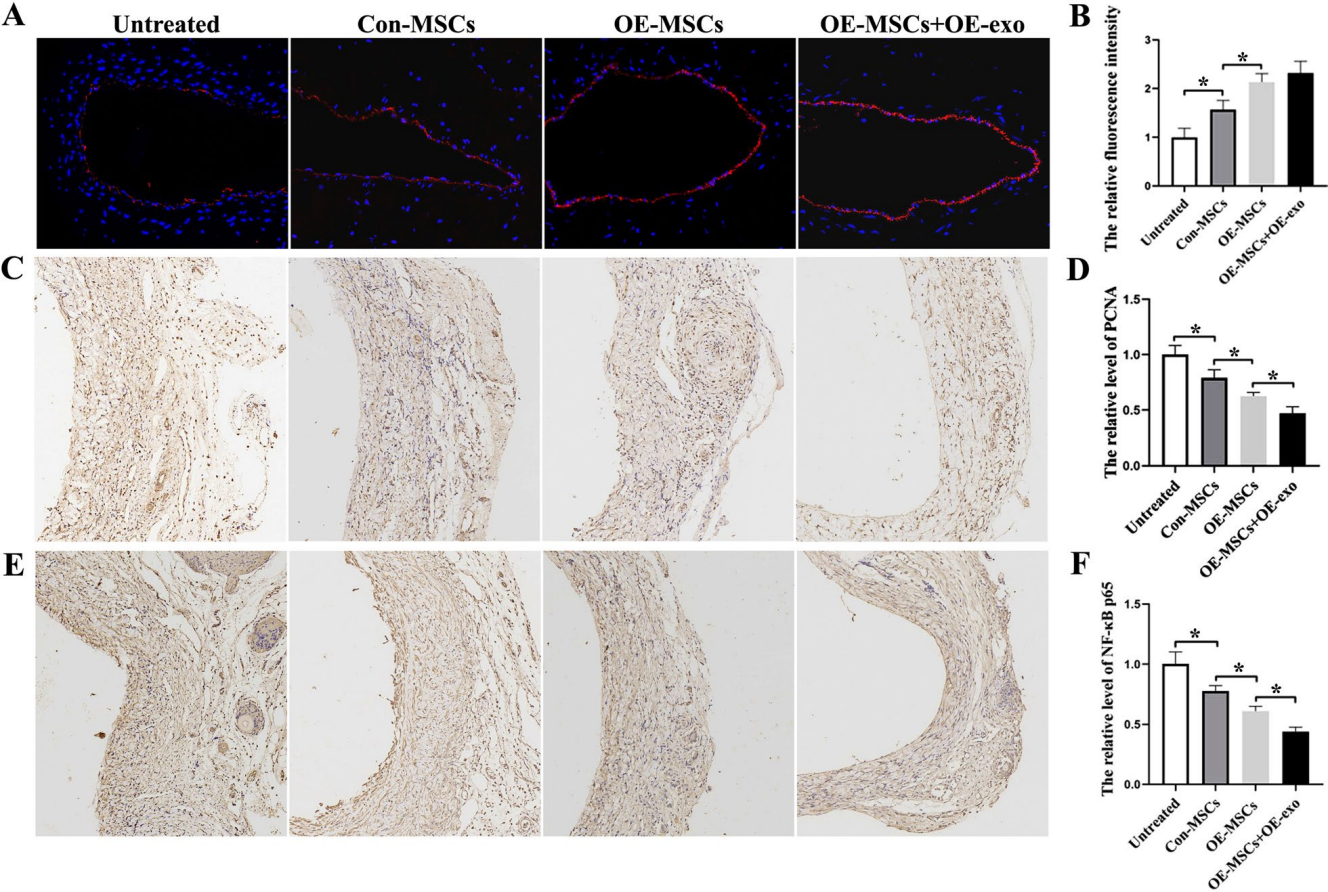
Evaluation of endothelial integrity and inflammatory proliferation of vein graft. **A** Representative image of vein graft stained with CD31. **B** Quantitative analysis of the relative fluorescence intensity (*p < 0.05). **C** Representative image of vein graft stained with PCNA. **D** Quantitative analysis of **C** (*p < 0.05). **E** Representative image of vein graft stained with NF-κB P65. **F** Quantitative analysis of **E** (*p < 0.05)

Endothelial integrity of the vessel wall was detected using CD31 staining. The results showed that endothelial integrity was impaired in the untreated group. Endothelial integrity was relatively better in the OE-MSCs + OE-exo group (Fig. 9A, B). Besides, proliferation and inflammation were assessed using PCNA and NF-κB P65 immunohistochemistry. The results showed that OE-MSCs + OE-exo administration significantly decreased the level of PCNA-positive cells (Fig. 9C, D) and NF-κB P65-positive cells (Fig. 9E, F). These results confirmed that OE-MSCs + OE-exo could effectively attenuate vein graft intimal hyperplasia by protecting endothelial integrity, synchronously inhibiting cell proliferation and inflammatory infiltration.

## Discussion

MSCs transplantation has been widely used to prevent atherosclerosis and promote angiogenesis in ischemic tissues [22, 23]. Using the autologous vein transplantation model in rats, we have confirmed that transplanted MSCs could home to the intima of the grafted vein, repair vascular endothelium and inhibit vascular remodeling [11]. However, some studies have reported that the homing or survival rate of transplanted MSCs was low, and the differentiation was uncertain [24, 25]. Therefore, we modified and regulated MSCs with lncRNA to improve the migration and survival of MSCs, improving the therapeutic effect of MSCs transplantation.

Increasing evidence has confirmed that lncRNA exerts suppressive or promotion effects regulating various biological processes. MIR155HG plays an essential role in hematopoiesis, inflammation, and tumorigenesis [26–28]. To explore the biological activity of MIR155HG, we constructed MIR155HG up-regulated or down-regulated MSCs. The western blot results showed that MIR155HG could promote NF-κB P65 phosphorylation, suggesting that the NF-κB pathway was involved in MSCs regulation by MIR155HG. Our previous experiments have confirmed that TNF-α promoted the survival and migration of MSCs under oxidative stress via the NF-κB pathway. Further, one study reported that NF-κB/MIR155HG had a mutual regulating relationship [29].

Through a series of cell function experiments, we found that OE-MIR155HG promoted cell viability, proliferation, and migration of MSCs. These biological effects were all regulated by the NF-κB pathway. H_2_O_2_-induced oxidative stress led to significant apoptosis of MSCs. OE-MIR155HG could relieve apoptosis significantly. This may be related to the up-regulation of pro-survival factors in MSCs. The experiments showed that OE-MIR155HG significantly elevated the level of HGF and VEGF in the supernatant, confirming our hypothesis. The BMS-345541 antagonizing NF-κB pathway reversed the protective role of OE-MIR155HG. Through these experiments, we found that MIR155HG was an important lncRNA with a wide range of regulatory effects. MIR155HG could significantly improve cell function in many directions, including proliferation, migration, secretion, and anti-apoptosis.

MSCs are the preferable source of therapeutic exosomes [30]. Exosomes have been reported to play a significant role in the paracrine effects. Exosomes are capable of delivering genetic materials. We observed that the RNA level of MIR155HG in exosomes derived from MIR155HG-MSCs was 4.9-fold higher than that of the control-MSCs. Similar genetic material changes were observed in exosomes as in transfected MSCs. In addition, the level of MIR155HG in HUVECs treated with OE-exo was 2.1-fold higher than that of con-exo. The results confirmed that exosomes derived from MSCs could efficiently deliver genetic materials into HUVECs. In our experiment, OE-exo could promote the migration of HUVECs and alleviate its apoptosis. Besides, in the sh-exo group, the activity of HUVECs was reduced, and apoptosis was increased. The exosomes could transmit the overexpressed genetic materials into target cells. One study reported that MSCs-derived exosomal MALAT1 could be transferred to osteoblasts and alleviate the symptoms of osteoporosis [31]. Therefore, we assume that it was not MIR155HG itself in sh-MIR155HG exosomes that affected the activity of HUVECs. Various miRNAs up-regulated by the ce-RNA mechanism may be the key to playing this role in sh-exo. There was sparse evidence on this. Through gene sequencing, we found that several miRNAs, including miR-133b, miR-206 and miR-675-3p, had significant negative feedback relationships with MIR155HG. These miRNAs could inhibit cell migration and promote apoptosis [32–34]. We will continue to explore this regulatory mechanism in subsequent studies.

We constructed the rat model of autogenous vein transplantation to simulate the process of intima hyperplasia in patients undergoing CABG. We found that intima hyperplasia was significantly alleviated through injecting both OE-MSCs and OE-exo into autologous vein grafted rats. The number of OE-MSCs homed to the intima of vein graft was significantly increased compared to the Con-MSCs group. The result suggested that OE-MSCs had a better home function in vivo. Part of the reason may be that MIR155HG enhances the migration ability of MSCs. Another reason may be that MIR155HG alleviates the apoptosis of MSCs in vivo. The fluorescence intensity of CD31 was higher in the OE-MSCs + OE-exo group than Con-MSCs. Furthermore, the enhanced vascular CD31 expression indicated greater intima integrity. Besides, the immunohistochemical test suggested that the expression of PCNA and NF-κB p65 in the vascular wall were significantly reduced in the OE-MSCs + OE-exo group than Con-MSCs or OE-MSCs group. The NF-κB played an essential role in inflammation and cell proliferation [35, 36]. These data confirmed that OE-MSCs + OE-exo could alleviate intimal hyperplasia by reducing cell proliferation and inflammatory response. The effect was better than using OE-MSCs alone. At present, no articles have been published about the combined application of functionally improved stem cells and exosomes for treating intimal hyperplasia. We believe that this synergistic effect could achieve better therapeutic outcomes.

## Conclusion

In summary, we confirmed that lncRNA-MIR155HG could promote the proliferation, migration, secretion, and anti-apoptosis of MSCs. Meanwhile, the NF-κB pathway participated in the regulation process. Exosome derived from MIR155HG-MSCs could delivery MIR155HG into endothelial cells and further inhibit endothelial cell apoptosis. Combining MIR155HG-MSCs and exosomes rich in MIR155HG could play a synergistic role in attenuating vein graft intimal hyperplasia more effectively. Then, we could provide a theoretical basis for improving the surgical treatment of coronary heart disease.

## Data Availability

The data that support the findings of this study are available from the corresponding authors upon reasonable request.

## Abbreviation

MSCs: Mesenchymal stem cells
lncRNA: Long non-coding RNA
OE-MSCs: MSCs with overexpressed MIR155HG
OE-exo: MIR155HG-MSCs-derived exosomes
HUVECs: Human umbilical vein endothelial cells
CABG: Coronary artery bypass grafting
MIR155HG: MiR-155 host gene
DMEM: Dulbecco's modified eagle medium
ECM: Endothelial cell medium
GFP: Green fluorescent proteins
MOI: Multiplicity of infection
PVDF: Polyvinylidene difluoride
VEGF: Vascular endothelium growth factor
HGF: Hepatocyte growth factor
VG: Van Gieson
PCNA: Proliferating cell nuclear antigen
IOD: Integral optical density
